# Duffing-type oscillator under harmonic excitation with a variable value of excitation amplitude and time-dependent external disturbances

**DOI:** 10.1038/s41598-021-82652-z

**Published:** 2021-02-03

**Authors:** Wojciech Wawrzynski

**Affiliations:** Department of Ship Operation, Faculty of Navigation, Gdynia Maritime University, Aleja Jana Pawła II 3, 81-345 Gdynia, Poland

**Keywords:** Engineering, Mathematics and computing, Physics

## Abstract

For more complex nonlinear systems, where the amplitude of excitation can vary in time or where time-dependent external disturbances appear, an analysis based on the frequency response curve may be insufficient. In this paper, a new tool to analyze nonlinear dynamical systems is proposed as an extension to the frequency response curve. A new tool can be defined as the chart of bistability areas and area of unstable solutions of the analyzed system. In the paper, this tool is discussed on the basis of the classic Duffing equation. The numerical approach was used, and two systems were tested. Both systems are softening, but the values of the coefficient of nonlinearity are significantly different. Relationships between both considered systems are presented, and problems of the nonlinearity coefficient and damping influence are discussed.

## Introduction

The issue of nonlinear oscillations has been extensively studied for years^1–5^. Generally, there are three reasons for this. First, nonlinear oscillations concern many physical systems. Second, the phenomena can be observed during nonlinear oscillations. Finally, the topic of chaotic response is relevant^6–9^.

To the issue of nonlinear oscillations, the Duffing equation, called the Duffing oscillator, is inseparably connected. Moreover, the Duffing oscillator is regarded as one of the prototypes for systems of nonlinear dynamics^10^. In mechanics, the Duffing-type equation in its basic form can be considered as a mathematical model of motion of a single-degree-of-freedom system with linear or nonlinear damping and nonlinear stiffness, e.g., this equation can be used for the case of a mass, suspended on a parallel combination of a dashpot with constant damping and a spring with nonlinear restoring force, excited by a harmonic force^11^. It can also be used to describe one or several degrees of freedom of the complex systems, e.g., the Duffing-type equation matches the general form of the equation of ships rolling^12^. Notwithstanding, the equation of the Duffing-type oscillator has a much wider application. For this reason, phenomena that are characteristic of nonlinear oscillations are often presented and discussed on the basis of this equation.

The literature related to the subject of nonlinear oscillations is extremely wide. We can find numerous books as well as many studies published in scientific journals where some of these journals are devoted practically only to the mentioned topics. In books, a comprehensive description of the issue is presented, and different methods used to solve and understand the behavior of nonlinear systems are discussed and compared^10,13–15^. In the studies published in scientific journals, a specific and narrowly defined problem is usually presented, which very often is combined with the proposition of a new solution method^16–19^.

Generally, in the studies concerning nonlinear systems or the Duffing equation directly, an analytical^11,20,21^ or/and numerical^12,22,23,43^ approach is applied. When the analytical approach is applied, the harmonic balance method (HBM) is most often used^3,4,17,23–25^. Among other methods, the iteration method^25–27^, method of multiple scales^28^, perturbation method^29,30^ or homotopy perturbation method^31,32^ can be mentioned. Very often, during research, the methods are combined^23^. Experiments are sometimes conducted to confirm the solution that is proposed or to provide credibility to the behavior of the mathematical model^33^. For example, in study^5^, a combination of experimental, analytical and numerical methods is used.

From tools that are used in the analysis of nonlinear systems, the most general tools are the frequency response curve^4,5,11,15,17–21, 27,33,34,38^, the backbone curve^11,12,19^ and, when the numerical approach is used, time histories^16,35,43^. In the group of more sophisticated tools, however, more specific techniques can also be mentioned: phase portraits^7,8,35–37^, bifurcation diagrams^8,38,39^, basins of attraction^40^ and Poincaré maps^8,35,41,42^.

Among the tools mentioned above, the tool that is practically always used in the analysis of nonlinear systems is the frequency response curve. Generally, this tool is used to describe the behavior of the system in the frequency domain (response to the excitation). More specifically, it is used to indicate the frequency of resonance and the frequency and amplitude of jump-up and jump-down phenomena. Unfortunately, such description of the system may be insufficient. The problem is the constant value of the amplitude of the excitation force/moment, which is used when developing the frequency response curve. In the more complex systems, the value of the excitation amplitude varies in time, and time-dependent external and internal disturbances can appear.

In view of the problem pointed out above, a new tool for the analysis of nonlinear systems is proposed. This new tool is the chart of bistability areas and areas of unstable solutions of the nonlinear system. It provides decidedly more comprehensive information about the considered system than the frequency response curve. In this study, this new tool is tested and discussed on the basis of the classic Duffing oscillator. During the analysis, the numerical approach is used, and two systems are tested. Both systems are softening systems, but the values of the coefficient of nonlinearity are significantly different. Relationships between both considered systems are presented, and problems of the nonlinearity coefficient and damping influence are discussed. Relationships between the frequency response curve and the chart of bistability areas and area of unstable solutions are also shown.

The standard Duffing equation can be written in the form:

1 $$\ddot{x} + \delta \dot{x} + \alpha x + \beta x^{3} = \xi cos\left( {\omega t} \right)$$

where *δ* is the damping coefficient, *α* and *β* are stiffness (restoring) coefficients, *ξ* is the coefficient of excitation, *ω* is the frequency of excitation and *t* is the time.

The stiffness, which is described by the formula *α x* + *β x*^3^, has a linear part controlled by *α* and a nonlinear part controlled by *β*. When *α* is > 0, then for *β* < 0, the stiffness characteristic is softening (Fig. 2), and for *β* > 0, it is hardening. When *β* = 0, then the Duffing equation describes a simple harmonic oscillator with linear damping (constant value of *δ*).

Usually, when the Duffing equation or equation similar to it is analyzed, the values of *δ*, *α*, *β* and *ξ* are assumed to be constant. In such a case, the number of parameters can be reduced, and the equation is transformed to the nondimensional form:

2 $$\ddot{y} + 2\eta \dot{y} + y + \varepsilon y^{3} = cos\left( {\omega_{0} \tau } \right)$$

3 $${\text{where:}}\quad y = \frac{\alpha }{\xi }x,\quad \tau = \left( {\sqrt \alpha } \right)t,\quad \eta = \frac{\delta }{2\sqrt \alpha },\quad \varepsilon = \frac{{\beta \xi^{2} }}{{\alpha^{3} }},\quad \omega_{0} = \frac{\omega }{\sqrt \alpha }$$

On the left side of Eq. (2), only two parameters are left: the damping coefficient *η* in its new form and the *ε* coefficient. This second coefficient is commonly treated as the parameter that determines the nonlinearity of the system. Note that depending on the considered issue, the initial form of Eq. (1) and, consequently, the form of parameter (3), may vary slightly, not only in terms of the notation.

**Figure 1:**
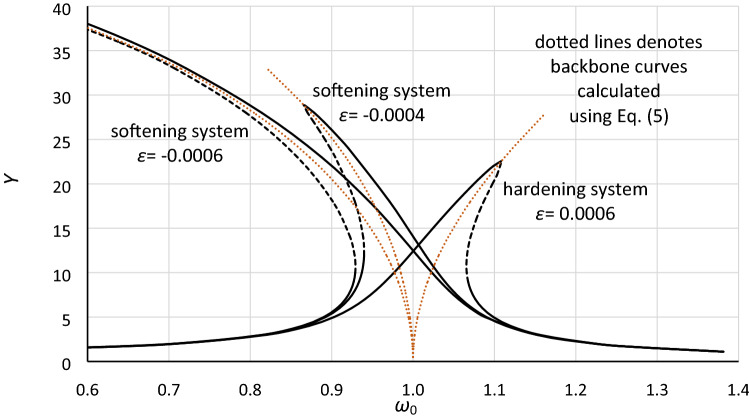
Frequency response curves for Eq. (4). Calculations were made for damping coefficient *η* = 0.02, nonlinearity coefficient *ε* = − 0.0006 and − 0.0004 (softening systems) and *ε* = 0.0006 (hardening system). Parts of the frequency response curves marked by dashed lines denote unstable solutions of the Duffing Eq. (1). Dotted lines denote backbone curves calculated using Eq. (5).

The reason for using the Duffing equation in the form (2) seems to be quite simple. This form of a nonlinear second-order differential equation is very convenient to provide some analytical solutions. For example, Brenan in^11^ applied the harmonic balance method (HBM) and assumed some simplifications to determine formula (4), which presents the frequency-amplitude relationship. Using this formula, the frequency response curves for the Duffing oscillator can be determined for both softening and hardening systems (Fig. 1), where the backbone curve is described by formula (5). Additionally, in^11^, Eq. (4) was used to find the solution for the amplitude of oscillations at the jump-up and jump-down frequencies as well as the solution for these frequencies.

4 $$\omega_{0;1,2} = \left( {1 + \frac{3}{4}\varepsilon Y^{2} \pm \left( {\frac{1}{{Y^{2} }} - 4\eta^{2} \left( {1 + \frac{3}{4}\varepsilon Y^{2} } \right)} \right)^{1/2} } \right)^{1/2}$$

5 $$\omega_{0;backbone} = 1 + \frac{3}{4}\varepsilon Y^{2}$$

Although analytical formulas are very convenient and useful, they have limitations. Formula (4) is restricted to only very small values of damping, and for a softening system with considerable values of stiffness nonlinearity, it gives incorrect solutions in the area of maximum possible oscillation amplitudes of the system. Moreover, formula (4) gives exact (steady-state) solutions in the area where Eq. (2) is unstable (dashed line in Fig. 1).

Another significant problem with analytical formulas is that all of them give only the steady-state solutions, disregarding the process of achieving it. For hardening systems, where the stiffness increases to infinity, this is not a problem, but for softening systems, it is. In dynamical systems, the stabilization of the response to the excitation is not immediate or directly proportional. In most cases, before the steady state is achieved, the system executes a few oscillations with amplitudes relative to the final and steady-state solution. If the steady-state solution is close to the maximum possible amplitude of oscillations (critical amplitude), then during oscillation stabilization, the critical amplitude can be exceeded and the steady-state solution not achieved; in the hardening systems, the amplitude of oscillations is unlimited. The problem of the stable solution is significant, especially when jump-up (bifurcation) occurs in the region marked as *A* in Fig. 4.

As mentioned above, all analytical solutions of the Duffing equation have limitations. Usually, these are associated with the system nonlinearity and the damping (which should be very small). In this paper, it will be shown how important damping is in the analysis of dynamical systems. In the theory of nonlinear oscillations, some relationships between the frequency response curves determined for different values of parameters of the Duffing equation can be found. Calculations presented in “Results of numerical simulations” section clearly show that these relationships are correct only when damping is equal or close to zero.

Note that for the constant value of damping, Eq. (2) can be and usually is analyzed in the frequency domain as the equation of only one parameter, the *ε* coefficient. In such analysis, the *ε* coefficient is used as the parameter that specifies/defines the nonlinearity of the system. This is discussable/questionable. The coefficient *ε* defined in form (3) is the product of *α*, *β* and *ξ,* where only the term *β*/*α*^3^ determines the degree of the system nonlinearity. The aggregation of the system nonlinearity and the amplitude of excitation means that we can obtain the same frequency response curve for different nonlinearities of the stiffness. Regardless of that, the real problem is quite different and is more substantial. In most physical oscillation systems, the amplitude of excitation (force or moment) usually varies over time, and some external and internal excitation impulses can occur. For example, in multi-degree-of-freedom systems, additional impulses of excitation may be derived from other degrees of freedom. In such cases, the frequency response curve (Fig. 1) is insufficient to describe the possible behavior of the nonlinear dynamic system, particularly with regard to the bifurcation phenomenon and jumps of amplitude accompanying it. This problem is independent of the use of Eqs. (1) or (2) and directly concerns the frequency response curve, which is commonly used.

## Form of the Duffing equation explored in the research

As noted above, using Eq. (2) and assuming a specified value of the damping coefficient, we obtain a single frequency response curve for an exact value of nonlinearity *ε* (Fig. 1). This can be read as a suggestion that oscillation systems with different combinations of *α*, *β* and *ξ* but the same value of *ε* can be described by a single frequency response curve. Unfortunately, this is not the case. When the phenomena that are characteristic of a nonlinear dynamical system (bistability, bifurcation, amplitude jumps and chaotic oscillations) are discussed, the frequency response curves (Fig. 1) do not show the differences between such systems. The form of these graphs cannot show the possible complexity of the behavior of the nonlinear system.

In most real dynamical systems, the amplitude of the excitation force or moment varies in time. Furthermore, in multi-degree-of-freedom systems, additional excitation impulses can appear. These additional impulses can have various values and are derived from degrees of freedom, which are coupled to the one being analyzed. To show the complexity of such systems behavior, a two-dimensional analysis is necessary. This analysis should be performed for the assumed surface of excitation-frequency values in combination with external disturbances taking the form of additional excitation impulses with a variable value of the force and a variable value of time duration. Moreover, the phase of oscillation is important when these additional impulses occur. Theoretically, to perform such an analysis and still have a nondimensional form of the Duffing equation, we can use a slightly modified Eq. (2):

6 $$\ddot{y} + 2\eta \dot{y} + y + \varepsilon y^{3} = \xi \left( \tau \right)cos\left( {\omega_{0} \tau } \right)$$

7 $${\text{where}}:\quad y = \alpha x,\quad \tau = \left( {\sqrt \alpha } \right)t,\quad \eta = \frac{\delta }{2\sqrt \alpha },\quad \varepsilon = \frac{{\beta { }}}{{\alpha^{3} }},\quad \omega_{0} = \frac{\omega }{\sqrt \alpha }$$

The coefficient of nonlinearity *ε* in the form (7) is now the product of *α* and *β* only, so we can say that now it truly determines only the degree of nonlinearity of stiffness. However, the specified value of coefficient *ε* (7) can still be obtained for various combinations of *α* and *β*. Therefore, to perform a comprehensive analysis, in this research, the Duffing equation in the almost standard form is explored:

8 $$\ddot{x} + 2\eta \dot{x} + \alpha x + \beta x^{3} = \xi \left( t \right)cos\left( {\omega t} \right)$$

For the system described by Eq. (8), the formula for the backbone curve, assuming the damping is close to 0, is:

9 $$\omega_{backbone}^{2} = \alpha + \frac{3}{4}\beta Y^{2}$$

During the presented research, numerical simulations were performed for two groups of coefficients of Eq. (8) (Table 1). These groups differ strongly in the value of the nonlinearity coefficient *ε*, which was calculated according to formula (7). For all cases in the first group, *ε* = − 0.0006, while for all cases in the second group, *ε* = − 0.045. Regardless of the differences in nonlinearity, the coefficients of the linear part of stiffness *α* are the same in both groups to obtain the corresponding cases.

**Table 1:** Coefficients of the Duffing equation that were tested during research (softening systems).

Case no	*α*	*β*	$\varepsilon = \frac{\beta }{{\alpha^{3} }}$	*x*_*cr*_	*x*_*max*_	*α·x*_*cr*_	*α·x*_*max*_
**GROUP A**							
A1	1.44225	− 0.0018	− 0.0006	28.30	16.34	40.82	23.57
A2	1.00000	− 0.0006	− 0.0006	40.82	23.57	40.82	23.57
A3	0.693361	− 0.0002	− 0.0006	58.87	33.99	40.82	23.57
**GROUP B**							
B1	1.44225	− 0.135	− 0.045	3.27	1.89	4.71	2.72
B2	1.00000	− 0.045	− 0.045	4.71	2.72	4.71	2.72
B3	0.693361	− 0.015	− 0.045	6.79	3.93	4.71	2.72

**Figure 2:**
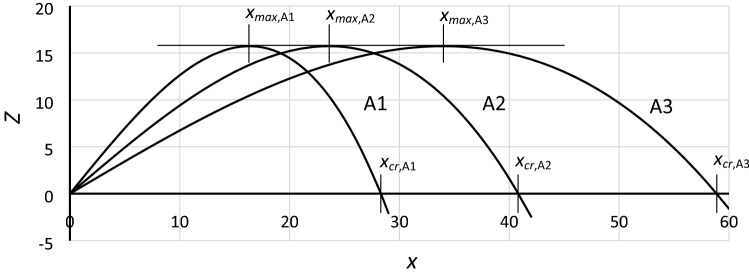
Curves of stiffness (softening stiffness) for cases in group **A** of Table 1.

Curves of stiffness for cases in group **A** are shown in Fig. 2 (units depend on the system that is considered). Some parameters of the curves and the relations between them are presented in Table 1. It is worthwhile to pay attention to the parameter *x*_*cr*_. This parameter determines the maximum possible amplitude of oscillations, which will be marked as *Y*_*cr*_ (critical amplitude).

## Oscillation bistability areas and area of unstable solution of the Duffing equation

**Figure 3:**
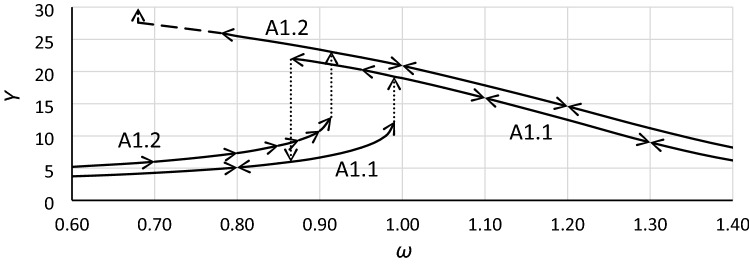
Frequency response curves for Eq. (8), case A1, for two values of excitation coefficient and damping coefficient *η* = 0.10.

When the Duffing equation in form (8) or (6) is explored, numerical methods are indispensable. Commonly, numerical simulations are performed for a wide range of excitation frequencies and a constant value of the excitation force. The typical effect of such calculations is the frequency response curve, as in Fig. 3 (in Fig. 3, two curves for two values of the excitation coefficient are shown). It is worth noting that the frequency response curves that are obtained are not exactly the same as in Fig. 1. Numerical methods do not give the possibility to appoint the part of curves marked by the dashed lines in Fig. 1—in this area, the solution of Eqs. (8) and (6) is unstable. In Fig. 3, dotted vertical lines show jumps up and jumps down (a phenomenon called bifurcation). In case A1.2, there is no jump-down (*Y*_*cr*_ is exceeded).

**Figure 4:**
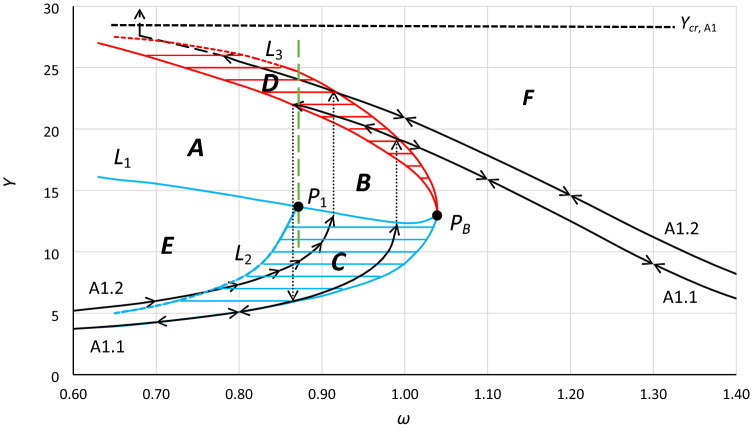
Frequency response curves (for two values of excitation coefficient) and oscillation bistability areas and area of unstable solution of the Duffing equation for case A1, calculated using Eq. (8) with damping coefficient *η* = 0.10.

The frequency response curves shown in Figs. 1 and 3 are insufficient to discuss the possible behavior of a dynamical system that is analyzed, especially when we wish to consider changes in the value of the excitation or the dynamics of the process of achieving the steady state solution. As stated above, to obtain a more comprehensive picture, a two-dimensional analysis should be performed. For each case presented in Table 1, numerical simulations were performed for the assumed surface of excitation-frequency values. The frequency of excitation was changed in the range including the backbone curve, while the amplitude of excitation was changed from 0 to the maximum value of a stable solution. The excitation coefficient was slowly increased, according to the *sin* function, from 0 to the target value during the early 800 s of each simulation—the change of this procedure can noticeably change the results that are obtained. Moreover, additional short impulses of excitation were generated (for simulations in the range of the bistability region) to force the transitions (amplitude jumps) between the nonresonant and resonant oscillations. The above sequences of calculations were carried out for a series of damping coefficients (*η* = 0.001, 0.01, 0.02, 0.05, 0.10, 0.15, 0.20, 0.25). All simulations were performed using the Runge–Kutta method for the same initial conditions ($\ddot{x}=0, \dot{x}=0$) and for the same time period (10,000 s). In the course of such calculations and data analysis, bistability areas and the area of unstable solutions of oscillations were determined in the form presented in Fig. 4.

In the following text, all parts of Fig. 4 are described. However, it should be noted that the meaning of the terms *area* and *region* differ. The term *area* refers to a separate part of the graph, while the term *region* means the frequency range. For example, in Fig. 4, *area B* is designated by the lower border of *area D*, line *L*_1_ and a dashed vertical green line at frequency *ω*_0_ = 0.87, whereas *region B* refers to frequencies between 0.87 and 1.04 rad/s. Furthermore, two types of bifurcation can be distinguished: the bifurcation due to the frequency change (pointed as *type F*) and due to the change of the excitation value (pointed as *type E*).

Point *P*_*B*_ is the bistability origin point. From this point, the development of bistability areas starts. Between bistability areas, the area of unstable solutions of the equation is located. In the analyzed system, jumps in oscillation amplitude (bifurcation phenomenon) can be observed only in the region of the area of unstable solutions. This region extends from the bistability origin point *P*_*B*_ towards lower frequencies for the system with softening stiffness and towards higher frequencies for the system with hardening stiffness.

Areas *C* and *D* are oscillation bistability areas. Area *C* is the area of nonresonant oscillations; however, the energy provided to the system is large enough for resonant oscillations within area *D*. Each point of area *C* is related to a second point within area *D*. Some scenarios of transitions between both areas were discussed in^12,43^. The lower limit of area *C* corresponds to the minimum value of the energy needed for resonant oscillations with the amplitude of the backbone curve (for damping close to zero).

The sum of areas *A* and *B* determines the complete area of unstable solutions of the equation that is analyzed. The upper and lower limits of these areas are the lines of bifurcations, type *F* and type *E*. This means that regardless of the parameter that is changing (excitation frequency or amplitude of excitation), entering into these areas causes a jump (bifurcation). Both areas of unstable solutions are separated by the vertical line at the frequency of point *P*_1_ (in Fig. 4, green dashed vertical line), which for systems with softening stiffness is located at the left-upper corner of area *C*. When bistability areas are not expanded enough, area *A* does not appear^12^. The behavior of the system on the border of areas *A* and *B* slightly differs. Entering area *B* through its lower limit causes a jump to the upper limit of area *D,* while entering it at its upper limit causes a jump to the lower limit of area *C*. In the case of area *A*, at its upper limit, the system behaves identically to the case of area *B*, while the lower limit of area *A* (line *L*_1_) not only is the line of bifurcation but also corresponds to the maximum value of excitation above which the system will jump over the critical amplitude *Y*_***cr***_ (Fig. 4). However, this concerns only cases when area *A* is entered directly from area *E*. Notwithstanding the above, stable oscillations for values of excitation amplitude exceeding the value that corresponds to the limit indicated by line *L*_1_ are possible^43^. For the case presented in Fig. 4, the following scenario can be executed. Let us assume that the system performs stable oscillations inside area *C* at the frequency *ω*_0_ = 0.80. With the use of the additional external impulse, oscillations can be transferred to area *D*. From this moment, the value of excitation can be increased to values over the limit pointed by line *L*_1_ (oscillations will exceed the upper limit of area *D*) but not over the value that corresponds to *Y*_*cr*_. It should be pointed out that the jump must be forced from area *C*, not from area *E*. Any attempt to force the jump from area *E* to *D* or over *D* will end up crossing critical amplitude *Y*_*cr*_.

Region *B* is the region where the oscillations that can have chaotic character are probable. This region extends from the frequency of *P*_*B*_ to the frequency of *P*_1_. The frequency response curve shown in Figs. 1 or 3 does not reveal this region completely. The highest probability of “chaotic” oscillations concerns frequencies close to *P*_*B*_. The reason is that in the pointed part of region *B,* the smallest values of the additional impulse are sufficient to force the jump between areas *C* and *D*. For case A1.1 (Fig. 3), we can identify only a part of bistability region *B* that extends from *ω*_0_ ≈ 0.88 to 0.99, while points *P*_1_ and *P*_*B*_ are undisclosed. Only a graph such as the one presented in Fig. 4 can reveal the whole region *B*.

Line *L*_1_ is the line of maximum amplitude of nonresonant oscillations that can be obtained at a given frequency, with a smooth increase in the value of excitation (starting from zero value). Inside region *A*, a further increase in the excitation induces bifurcation with jump-up over the critical amplitude *Y*_*cr*_. Inside region *B*, further increase of the excitation induces bifurcation with jump-up to the upper limit of area *D* and next above it.

Line *L*_2_ is the line of maximum amplitude of nonresonant oscillations at which it is possible to force a jump to the area of resonant oscillations—to the upper limit of area *D* (line *L*_3_). Determination of the exact course of both lines, *L*_2_ and *L*_3_, is the most problematic part of the process of determining bistability areas. The problem is to find the combination of the maximum possible amplitude of nonresonant oscillations and the value of the additional impulse and the phase of oscillations at which this impulse should occur to induce the jump-up with stable solution. In practically all analyzed cases, the final course of lines *L*_2_ and *L*_3_ (inside region *A*) have been extrapolated, and these parts of both lines are dashed (in all figures). In Fig. 6c, lines *L*_3_ and *L*_2_ are obtained directly from numerical simulations (solid line), and it can be seen that *L*_3_ is flattened. In many cases, this flattening is much more visible. Determining lines *L*_2_ and *L*_3_ is an issue for further research.

For any system where the value and/or frequency of excitation vary in time, the chart shown in Fig. 4 will definitely be better than the frequency response curve. In Fig. 4, it can be seen that the frequency response curves (curves marked as A1.1 and A1.2) appoint only single points at the boundary of the area of unstable solutions of the Duffing equation that is tested. Therefore, the frequency response curve reveals only two points of bifurcation, while the whole boundary of the area of unstable solutions is the line of the bifurcation phenomenon.

## Results of numerical simulations

All numerical simulations were performed with the use of the Duffing equation in the form (8) for the cases presented in Table 1. Simulations and data analysis were conducted with the use of the computer algebra systems (CAS) *Mathematica* and MATLAB.

**Figure 5:**
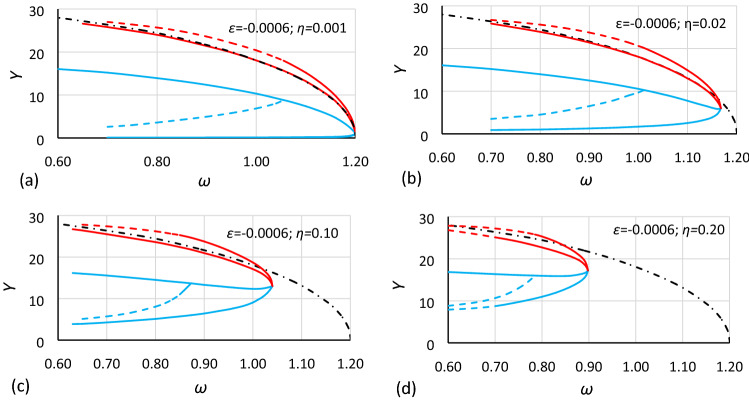
Bistability areas and area of unstable solution of the Duffing equation for nonlinearity *ε* = − 0.0006. Case A1 (*α* = 1.44225; *β* = − 0.0018) for different values of damping coefficient *η*: (**a**) 0.001, (**b**) 0.02, (**c**) 0.10, (**d**) 0.20. The dashed-dotted line is the backbone curve calculated using Eq. (9).

**Figure 6:**
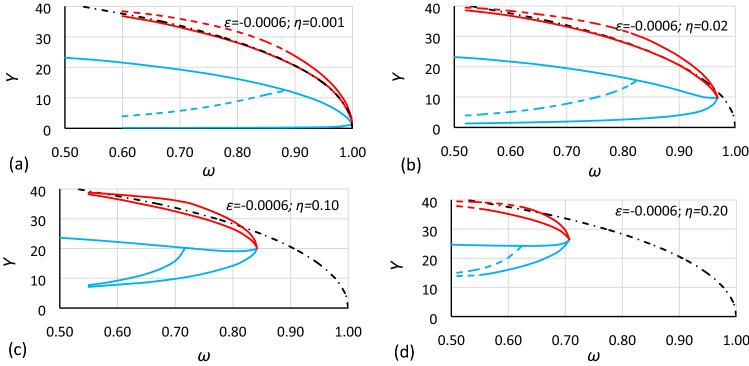
Bistability areas and area of unstable solution of the Duffing equation for nonlinearity *ε* = − 0.0006. Case A2 (*α* = 1.000; *β* = − 0.0006) for different values of damping coefficient *η*: (**a**) 0.001, (**b**) 0.02, (**c**) 0.10, (**d**) 0.20. The dashed-dotted line is the backbone curve calculated using Eq. (9).

**Figure 7:**
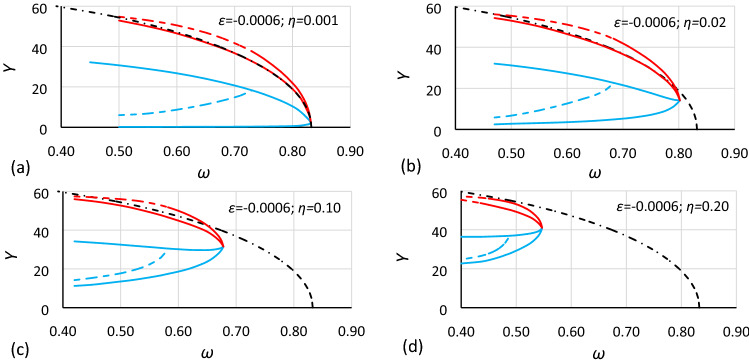
Bistability areas and area of unstable solution of the Duffing equation for nonlinearity *ε* = − 0.0006. Case A3 (*α* = 0.693361; *β* = − 0.0002) for different values of damping coefficient *η*: (**a**) 0.001, (**b**) 0.02, (**c**) 0.10, (**d**) 0.20. The dashed-dotted line is the backbone curve calculated using Eq. (9).

**Figure 8:**
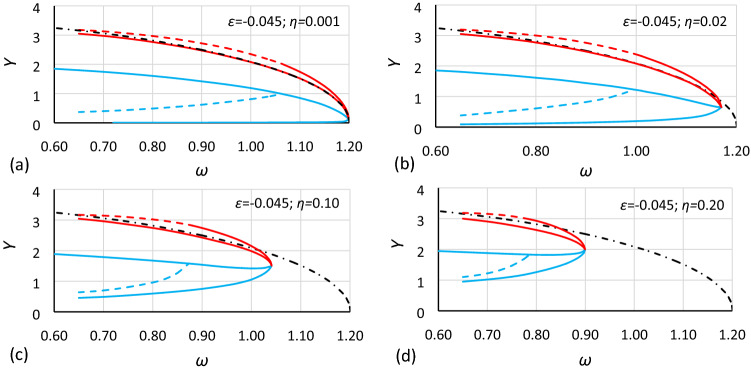
Bistability areas and area of unstable solution of the Duffing equation for nonlinearity *ε* = − 0.045. Case B1 (*α* = 1.44225; *β* = − 0.135) for different values of damping coefficient *η*: (**a**) 0.001, (**b**) 0.02, (**c**) 0.10, (**d**) 0.20. The dashed-dotted line is the backbone curve calculated using Eq. (9).

**Figure 9:**
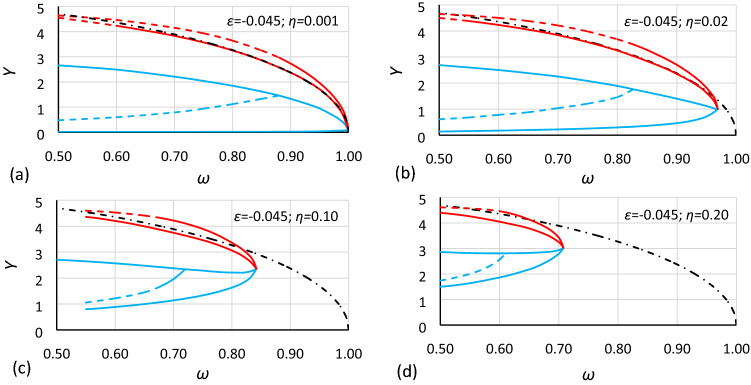
Bistability areas and area of unstable solution of the Duffing equation for nonlinearity *ε* = − 0.045. Case B2 (*α* = 1.000; *β* = − 0.045) for different values of damping coefficient *η*: (**a**) 0.001, (**b**) 0.02, (**c**) 0.10, (**d**) 0.20. The dashed-dotted line is the backbone curve calculated using Eq. (9).

**Figure 10:**
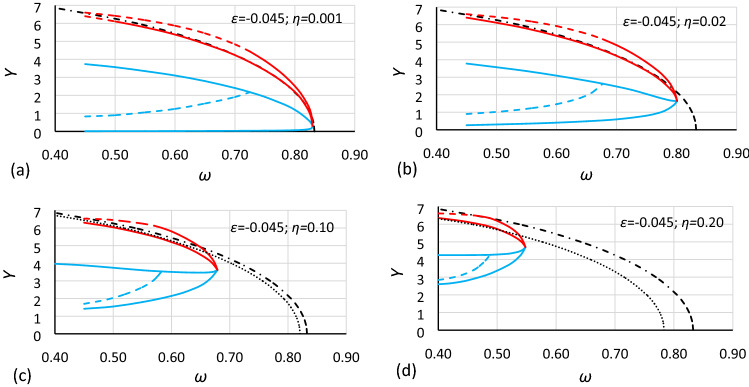
Bistability areas and area of unstable solution of the Duffing equation for nonlinearity *ε* = − 0.045. Case B3 (*α* = 0.693361; *β* = − 0.015) for different values of damping coefficient *η*: (**a**) 0.001, (**b**) 0.02, (**c**) 0.10, (**d**) 0.20. The dashed-dotted line is the backbone curve calculated using Eq. (9). The dotted line is the backbone curve calculated using Eq. (10).

Most of the basic data obtained from the numerical simulations are presented in two groups of drawings. The first group concerns the nonlinearity with *ε* = − 0.0006 (Figs. 5, 6 and 7), and the second group concerns the nonlinearity with *ε* = − 0.045 (Figs. 8, 9 and 10). Each group consists of 3 cases that have the same nonlinearity but differ in the value of *α* (the coefficient of linear part of stiffness). Finally, for each case, charts for four different values of damping coefficients are presented. Additionally, in each drawing, the backbone curve calculated according to Eq. (9) is plotted using the dashed-dotted line.

At first glance, it is clearly visible that damping has a large impact on the size and location of the bistability areas and the area of unstable solution of the Duffing equation. The initial analysis of drawings shows that:

- The general characteristics of all charts are similar.
- For larger values of damping, areas of bistability and area of unstable solution are decreasing.
- For larger values of damping, areas of bistability, areas of unstable solution and the bistability origin point are moving towards smaller values of frequency (for softening systems) and larger values of oscillation amplitude.
- For smaller values of the *α* coefficient, the areas of bistability and the area of unstable solution decrease.
- For the same values of *α* and *η* but different nonlinearities (different values of *ε* coefficient), the frequencies of the bistability origin point seem to be equal (comparison of Figs. 5 and 8, Figs. 6 and 9, and Figs. 7 and 10).
- For the same values of *α* and *η* but different nonlinearities, areas of bistability and area of unstable solution look very similar (comparison of Figs. 5 and 8, Figs. 6 and 9, and Figs. 7 and 10). The only difference seems to be the values on the axis of oscillation amplitude.
- The backbone curve overlaps with the lower limit of area *D,* so it can be stated that the backbone curve determines the lower limit of the area of resonant oscillations. A good coincidence of both lines can be observed only for very small values of damping. The reason is that in formula (9), as in formula (5), damping is not included. To a certain extent, the backbone curve can be corrected due to damping, according to the known rule (10). However, this solution is not fully correct, as shown in Fig. 10, where the corrected backbone curve (dotted black curve) is placed for cases with damping coefficients of 0.10 and 0.20.
  10 $$\omega_{backbone,corr}^{2} = \omega_{backbone}^{2} - 2\eta^{2}$$

It is generally accepted that the frequency response curves calculated for the same coefficient of nonlinearity can be scaled/converted accordingly to simple rules:

11 $$\omega_{0} = \frac{\omega }{\sqrt \alpha },\quad Y_{0} = \alpha Y$$

**Figure 11:**
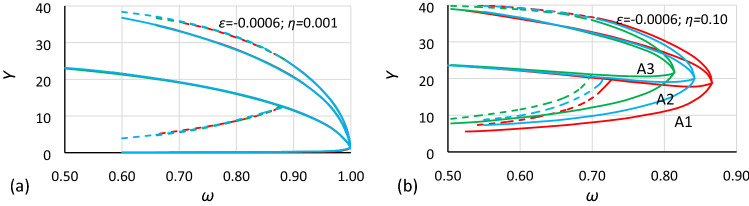
Comparison of oscillation bistability areas and areas of unstable solution for nonlinearity *ε* = − 0.0006, for different values of damping coefficient *η*: (**a**) 0.001, (**b**) 0.10. Comparison of case A2 (blue; *α* = 1.0000) with cases A1 (red; *α* = 1.44225) and A3 (green; *α* = 0.693361) scaled to A2 according to rules (11).

**Figure 12:**
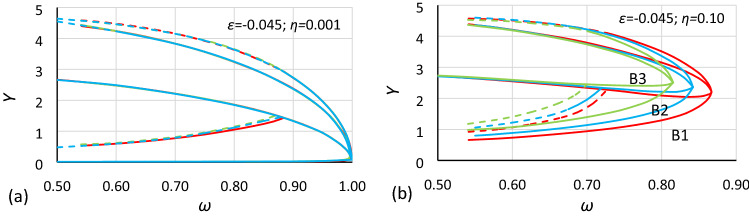
Comparison of oscillation bistability areas and areas of unstable solution for nonlinearity *ε* = − 0.045, for different values of damping coefficient *η*: (**a**) 0.001, (**b**) 0.10. Comparison of case B2 (blue; *α* = 0.1.0000) with cases B1 (red; *α* = 1.44225) and B3 (green; *α* = 0.693361) scaled to B2 according to rules (11).

These rules should also apply in the case of bistability areas. Comparisons made in Figs. 11 and 12 clearly show that rules (11) work only when the value of damping is very small, in fact only when damping is close to zero. Regardless of that, a comparison of Figs. 11b and 12b show that for the same value of damping, the displacements between the corresponding charts are similar for both cases (for other values of damping, the effect is the same). If so, it can be assumed that the bistability area dependence on damping is the same for different values of nonlinearity. Nevertheless, *α* as well as the amplitude of oscillations should be taken into account. Both formulas (11) should be extended to include these dependences.

As mentioned earlier, it is easy to see in Figs. 5, 6, 7, 8, 9 and 10 that for the same values of *α* and *η* but different nonlinearities, areas of bistability and areas of unstable solution look very similar and that the frequencies of the bistability origin point seem to be equal. The difference applies only the *Y* axis. To test this relationship, the values of amplitude should be scaled. Unfortunately, scaling in accordance with rule (11) was not effective. It was found that the values of amplitude should be recalculated to a nondimensional form according to the formula:

12 $$Y_{n} = \frac{Y}{{Y_{cr} }}$$

where *Y*_*cr*_ is the critical amplitude of oscillation:

13 $$Y_{cr} = \sqrt {\left| {\frac{\alpha }{\beta }} \right|}$$

**Figure 13:**
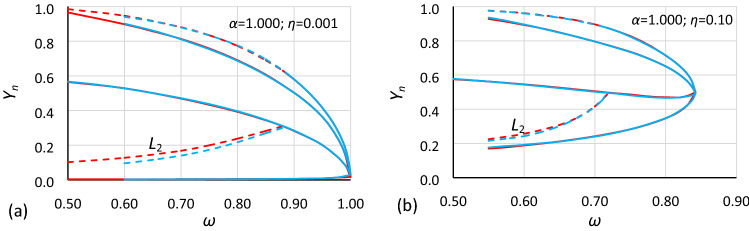
Comparison of oscillation bistability areas for different nonlinearities: *ε* = − 0.045 (red) and *ε* = − 0.0006 (blue) for the same value of the *α* coefficient (cases A2 and B2: *α* = 1.000) for two different values of the damping coefficient *η*: (**a**) 0.001, (**b**) 0.10 and *Y* values recalculated to nondimensional form according to formula (12).

**Figure 14:**
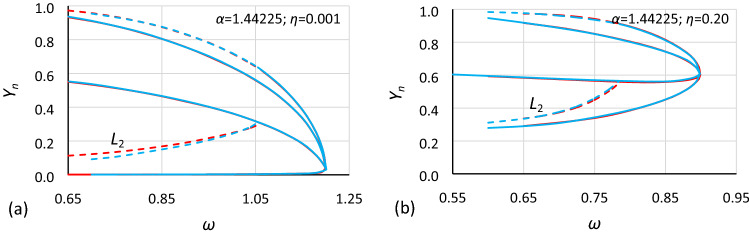
Comparison of oscillation bistability areas for different nonlinearities: *ε* = − 0.045 (red) and *ε* = − 0.0006 (blue) for the same value of the *α* coefficient (cases A1 and B1: *α* = 1.44225) for two different values of the damping coefficient *η*: (**a**) 0.001, (**b**) 0.20 and *Y* values recalculated to nondimensional form according to formula (12).

In Figs. 13 and 14, the use of formula (12) is shown. It is clearly visible that for the same value of *α* and *η* after scaling *Y* values according to rule (12), charts of bistability areas determined for different nonlinearities become identical. It should be highlighted that the difference between the nonlinearity of the compared cases is very large, the coefficients of nonlinearity have values of − 0.0006 and − 0.045. If there are some discrepancies between the charts, they apply only to lines *L*_2_ and are the consequence of the extrapolation procedure that was used to determine them. Additionally, rule (12) is independent of damping (in Fig. 13*η* = 0.10, and in Fig. 14*η* = 0.20).

**Figure 15:**
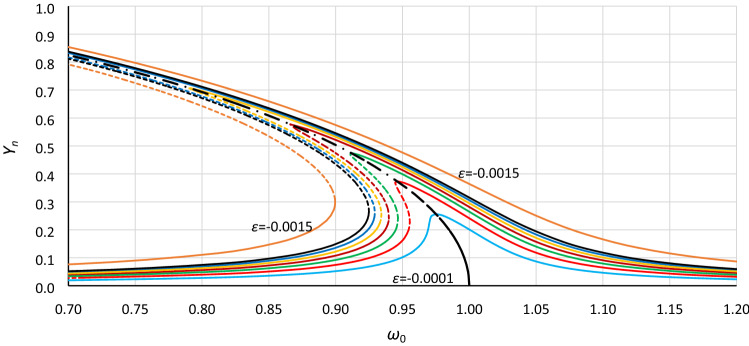
Frequency response curves calculated according to Eq. (4) for a series of values of the nonlinearity coefficient *ε* (0.001-lowest curve, 0.002, 0.003, 0.004, 0.005, 0.006, 0.007, 0.015-highest curve) and the constant value of damping coefficient *η* = 0.02 and scaled according to formula (12) for values of *Y*_*cr*_ calculated assuming that *α* = 1 and *ξ* = 1. The dashed-dotted line is the backbone curve; Eq. (14).

**Figure 16:**
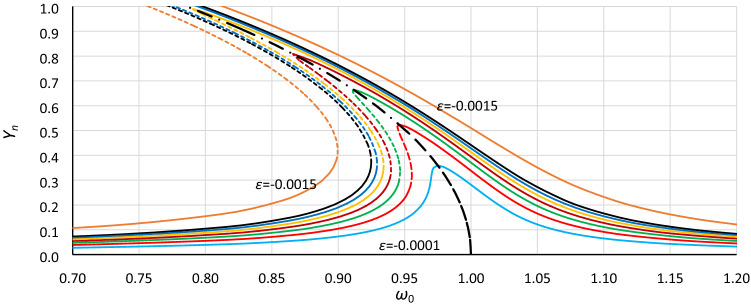
Frequency response curves calculated according to Eq. (4) for a series of values of the nonlinearity coefficient *ε* (0.001-lowest curve, 0.002, 0.003, 0.004, 0.005, 0.006, 0.007, 0.015-highest curve) and the constant value of damping coefficient *η* = 0.02 and scaled according to formula (12) for values of *Y*_*cr*_ calculated assuming that *α* = 1.40 and *ξ* = 1. The dashed-dotted line is the backbone curve; Eq. (14).

Next, formula (12) is applied to convert the frequency response curves calculated with the use of formula (4) (similar to those presented in Fig. 1). The effect of such calculations is presented in Figs. 15 and 16. The frequency response curves were calculated for a series of values of the nonlinearity coefficient *ε* and the constant value of damping coefficient *η*. Next, obtained curves were scaled according to formula (12), where values of *Y*_*cr*_ were calculated assuming for each value of *ε* that *ξ* = 1 and *α* = 1 (Fig. 15) and *α* = 1.40 (Fig. 16). In these cases, the effect of calculations is not a single curve but a series of curves. This is because the constant value of the coefficient of excitation *ξ* gives different response curves for different *ε* and *η* > 0. Nevertheless, we can clearly see the convergence of the upper and lower branches of each curve relative to the backbone curve, which was calculated according to formula (5), where the coefficient of nonlinearity *ε* has been replaced by coefficient *α*:

14 $$\omega_{0;backbone} = 1 + \frac{3}{4}\alpha Y^{2}$$

Figures 13 and 14 and particularly Figs. 15 and 16 show that the commonly used coefficient of nonlinearity *ε* in the form (3) as well as in the form (7) differentiates/divides/groups cases of the Duffing equation not entirely in a correct way, so the coefficient *ε* is an ambiguous parameter. The crucial parameter is the *α* coefficient, where the *β* coefficient determines only the value of the critical amplitude of oscillations. When the individual/selected cases are tested, the *ε* coefficient can be very convenient, but when a general analysis is made, this coefficient is insufficient.

Conversion between the charts calculated for different values of the *α* coefficient but the same value of damping is very simple. The relationship is defined by the following formula:

15 $$\alpha_{1} \cdot Y_{cr1} = \alpha_{2} \cdot Y_{cr2}$$

It was tested that using formula (15), the frequency response curves presented in Fig. 15 can be converted into those in Fig. 16. The new set of drawings concerns a new value of the *α* coefficient at the same value of damping. Notwithstanding the above, Fig. 15 and especially 16 show that analytical formula (4) has a significant problem with the curve convergence for larger values of the *ε* coefficient when the oscillation amplitude tends to *Y*_*cr*_ (in Figs. 15 and 16 when *Y* tends to 1) This problem increases for smaller values of damping as well as for larger values of *α*. Generally, any analytical solution of a nonlinear differential equation has larger or smaller limitations.

The calculations and comparisons that were made above show that for the standard Duffing Eq. (1), one set of charts (calculated for series values of the damping coefficient) such as those in Figs. 13 or 14 is enough to describe it. One set of drawings can be converted to another set, for any combination of *α* and *β* with only one reservation: that the damping coefficients *η* are the same. At the current stage of the research that is being developed, damping seems to be the most problematic parameter. In Figs. 11b and 12b, it is visible that the damping induces a horizontal and vertical shift. Additionally, it can be stated that a shift due to damping is independent from the *ε* parameter.

## Application

As an example of application of the chart of bistability areas and area of unstable solutions for the nonlinear system, the problem of ship rolling can be discussed. Rolling is a phenomenon adversely affecting the operations of a ship. Under certain conditions, the amplitude of rolling can exceed angles of 0.6 rad and sometimes even 0.7 rad. Such large amplitudes of rolling can be a serious threat to the safety of ships, cargo and people.

Numerical simulations were conducted with the 1DOF ship rolling model (the Duffing-type equation) with damping dependent on amplitude and frequency^43^:

16 $$\ddot{\phi } + 2\alpha \left( {\omega_{e} } \right) \cdot \dot{\phi } + \beta \left( {\omega_{e} } \right) \cdot \dot{\phi }\left| {\dot{\phi }} \right| + \gamma \left( {\omega_{e} } \right) \cdot \dot{\phi }^{3} + \frac{g}{{r_{x}^{2} }}GZ\left( \phi \right) = \xi_{w} \left( t \right) \cdot cos\left( {\omega_{e} t} \right)$$

where *r*_*x*_ is the gyration radius of a ship and added masses, *GZ* is the restoring arm and *ξ*_*w*_ is the time-dependent exciting moment coefficient. Damping coefficients *α*, *β* and *γ* for the exact frequency were calculated analytically according to the simple Ikeda method^44^ with modification discussed in^45^ and next individually fitted for the 4^th^-order polynomial with the roll frequency as an argument^43^:

17 $$\alpha \left( \omega \right) = a_{\alpha } + b_{\alpha } \omega + c_{\alpha } \omega^{2} + d_{\alpha } \omega^{3} + e_{\alpha } \omega^{4}$$

18 $$\beta \left( \omega \right) = a_{\beta } + b_{\beta } \omega + c_{\beta } \omega^{2} + d_{\beta } \omega^{3} + e_{\beta } \omega^{4}$$

19 $$\gamma \left( \omega \right) = a_{\gamma } + b_{\gamma } \omega + c_{\gamma } \omega^{2} + d_{\gamma } \omega^{3} + e_{\gamma } \omega^{4}$$

The arm of the restoring moment was approximated by a ninth-order polynomial with odd powers only:

20 $$GZ\left( \phi \right) = C_{1} \cdot \phi + C_{3} \cdot \phi^{3} + C_{5} \cdot \phi^{5} + C_{7} \cdot \phi^{7} + C_{9} \cdot \phi^{9}$$

**Figure 17:**
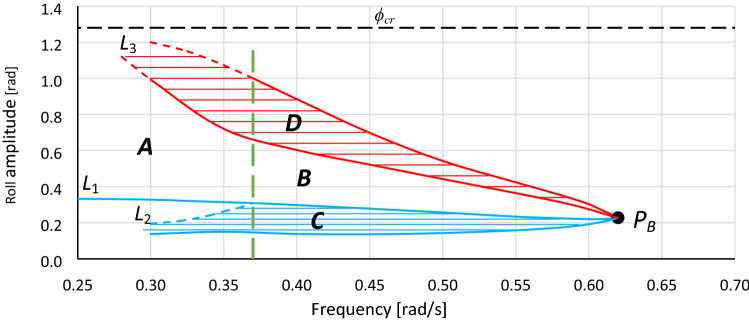
Bistability areas and area of unstable solution of roll Eq. (17) for the offshore support vessel (*T* = 6.10 m; the GZ curve represents a softening spring), determined for the damping dependent on the rolling amplitude and frequency.

Numerical simulations were conducted with the use of Eq. (16) for an offshore support vessel (OSV) with a softening restoring moment. The chart of bistability areas and area of unstable solutions that have been determined is shown in Fig. 17. For the presented case, we should pay special attention to area C (the area of nonresonant rolling). This area has a considerable width and is located at roll amplitudes (0.15–0.30 rad), which are often observed during adverse weather conditions. Such roll amplitudes are unfavorable but do not cause a real threat and can be considered typical for the mentioned conditions. However, the energy provided to the system is large enough for resonant rolling within area *D*. The transition to area *D* can be caused by a group of higher waves, wind or impulse from the coupled DOFs^43^. Inside area *D,* the possible amplitudes of rolling are much larger than inside area *C*. At frequency 0.45, the amplitude of resonant rolling, which corresponds to the upper limit of area C, is close to 0.7 rad (40°)* and such amplitudes of rolling can be a real threat. Moreover, a large change in roll amplitude (from 0.3 to 0.7 rad) can be relatively rapid—for strong impulses, it can occur over 1–3 cycles. To reduce the chance of occurrence of such large and rapid changes in the rolling amplitude, the ship should change the course and/or speed (change wave encounter frequency) or change the loading condition parameters (e.g., by ballasting). Ideally, the ship would operate outside area *C*. Analyzing ship safety, we should take into account not one combination of rolling frequency and amplitude (at which bifurcation can occur) but the whole area C. This is due to the variability in the wave height, wave direction, wind parameters and the course of the ship.

It is clear that even such abbreviated analysis is impossible when the frequency response curve is used.

## Conclusions

In this paper, a new tool for the analysis of systems described by the Duffing-type equation is proposed. This new tool is the chart of bistability areas and area of unstable solutions of the Duffing equation. It was demonstrated that the frequency response curve provides insufficient information about a nonlinear system described by the equation in type of the Duffing oscillator. The frequency response curve can be considered sufficient only when the value of the excitation force/moment is constant. The proposed chart of bistability areas can be treated as an extension to the frequency response curve.

Additionally, the paper shows that the coefficient of nonlinearity *ε* in form (3) as well as in form (7) is an ambiguous parameter and differentiates/divides/groups cases of the Duffing equation not in an entirely correct way. For the Duffing equation with nonlinear stiffness described by the formula *α x* + *β x*^3^, the crucial parameter is the *α* coefficient, where the *β* coefficient governs only the value of the critical amplitude of oscillations. For two systems that differ even very strongly in the value of *ε* but have the same value of *α* when the *Y* values are recalculated according to formula (12), their areas of bistability and area of unstable solution become identical. This means that to describe any Duffing oscillator, only the *α* coefficient and damping coefficient are needed.

Finally, it is worth highlighting that the area of unstable solutions of the Duffing equation, especially the part that is close to the bistability origin point, may be directly related to the possibility of the occurrence of chaotic oscillations.

## References

[1] Duffing, G. Erzwungene Schwingungen bei Veränderlicher Eigenfrequenz und ihre Technische Bedeutung (Forced oscillators with variable eigenfrequency and their technical meaning). *F. Vieweg Sohn*, 41–42 (1918) **(in German)**.

[2] Hayashi C, Shepard S, Winkler I, Glenn S, Harris E, Quaid D, Hershey B, Kaufman P, Chartoff R, Wolfe T (1964). Nonlinear Oscillations in Physical Systems.

[3] Mickens R (1984). Comments on the method of harmonic balance. J. Sound Vib..

[4] Liu L, Thomas J, Dowell E, Attar P, Hall K (2006). A comparison of classical and high dimensional harmonic balance approaches for a Duffing oscillator. J. Comput. Phys..

[5] Agarwal V, Zheng X, Balachandran B (2018). Influence of noise on frequency responses of softening Duffing oscillators. Phys. Lett. A.

[6] Ackerhalt JR, Galbraith HW, Milonni PW, Mandel L, Wolf E (1984). Onset of chaos in Duffing oscillator systems. Coherence and Quantum Optics V.

[7] Leng XL, Wu CL, Ma XP, Meng G, Fang T (2005). Bifurcation and chaos analysis of stochastic Duffing system under harmonic excitations. Nonlinear Dyn..

[8] Zhang M, Yang J (2007). Bifurcations and chaos in Duffing equation. Acta Math. Appl. Sin. (Engl. Ser.).

[9] Lei Y, Fu R, Yang Y, Wang Y (2016). Dichotomous-noise-induced chaos in a generalized Duffing-type oscillator with fractional-order deflection. J. Sound Vib..

[10] Korsch J, Jodl H, Hartmann T (2008). Chaos.

[11] Brennan MJ, Kovacic I, Carrella A, Waters TP (2008). On the jump-up and jump-down frequencies of the Duffing oscillator. J. Sound Vib..

[12] Wawrzyński W (2018). Bistability and accompanying phenomena in the 1-DOF mathematical model of rolling. Ocean Eng..

[13] Nayfeh AH, Mook DT (2008). Nonlinear Oscillations.

[14] Kovacic I, Brennan MJ (2011). The Duffing Equation: Nonlinear Oscillators and Their Behaviour.

[15] Warminski J, Lenci S, Cartmell PM, Rega G, Wiercigroch M (2012). Nonlinear Dynamic Phenomena in Mechanics.

[16] Chen YM, Liu JK (2007). A new method based on the harmonic balance method for nonlinear oscillators. Phys. Lett. A.

[17] Grolet A, Thouverez F (2012). On a new harmonic selection technique for harmonic balance method. Mech. Syst. Signal Process..

[18] Taghipour J, Dardel M (2015). Steady state dynamics and robustness of a harmonically excited essentially nonlinear oscillator coupled with a two-DOF nonlinear energy sink. Mech. Syst. Signal Process..

[19] Londoño JM, Cooper JE, Neild SA (2017). Identification of systems containing nonlinear stiffnesses using backbone curves. Mech. Syst. Signal Process..

[20] Friswell MI, Penny JET (1994). The accuracy of jump frequencies in series solutions of the response of a Duffing oscillator. J. Sound Vib..

[21] Ho C, Lang Z, Billings S (2014). A frequency domain analysis of the effects of nonlinear damping on the Duffing equation. Mech. Syst. Signal Process..

[22] Kamiński M, Corigliano A (2015). Numerical solution of the Duffing equation with random coefficients. Meccanica.

[23] Guillot L, Vergez Ch, Cochelin B (2019). Continuation of periodic solutions of various types of delay differential equations using asymptotic numerical method and harmonic balance method. Nonlinear Dyn..

[24] Luo ACJ, Huang J (2011). Approximate solutions of periodic motions in nonlinear systems via a generalized harmonic balance. J. Sound Vib..

[25] Mickens R (1987). Iteration procedure for determining approximate solutions to non-linear oscillator equations. J. Sound Vib..

[26] Mickens RE (2005). A generalized iteration procedure for calculating approximations to periodic solutions of truly nonlinear oscillators. J. Sound Vib..

[27] Hoang T, Duhamel D, Foret G, Yin HP, Argoul P (2017). Frequency dependent iteration method for forced nonlinear oscillators. Appl. Math. Model..

[28] Starosta R, Sypniewska-Kaminska G, Awrejcewicz J (2017). Quantifying non-linear dynamics of mass-springs in series oscillators via asymptotic approach. Mech. Syst. Signal Process..

[29] Chen SH, Cheung YK, Lau SL (1991). On perturbation procedure for limit cycle analysis. Int. J. Non-Linear Mech..

[30] Leung AYT, Guo Z (2009). Homotopy perturbation for conservative Helmholtz-Duffing oscillators. J. Sound Vib..

[31] Cveticanin L (2006). Homotopy-perturbation method for pure nonlinear differential equation. Chaos Solitons Fractals.

[32] Beléndez A, Beléndez T, Márquez A, Neipp C (2008). Application of He’s homotopy perturbation method to conservative truly nonlinear oscillators. Chaos Solitons Fractals.

[33] Contento G, Francescutto A, Piciullo M (1996). On the effectiveness of constant coefficients roll motion equation. Ocean Eng..

[34] Jain S, Breunung T, Haller G (2019). Fast computation of steady- state response for high-degree-of-freedom nonlinear systems. Nonlinear Dyn..

[35] Ma SJ, Xu W, Fang T (2008). Analysis of period-doubling bifurcation in double-well stochastic Duffing system via Laguerre polynomial approximation. Nonlinear Dyn..

[36] Chen H, Huang D, Jian Y (2018). The saddle case of Rayleigh-Duffing oscillators. Nonlinear Dyn..

[37] Georgiev ZD, Uzunov IM, Todorov TG (2018). Analysis and synthesis of oscillator systems described by a perturbed double-well Duffing equation. Nonlinear Dyn..

[38] Miwadinou CH, Hinvi LA, Monwanou AV, Chabi Orou JB (2017). Nonlinear dynamics of a φ6−modified Duffing oscillator: resonant oscillations and transition to chaos. Nonlinear Dyn..

[39] Du L, Zhao Y, Lei Y, Hu J, Yue X (2018). Suppression of chaos in a generalized Duffing oscillator with fractional-order deflection. Nonlinear Dyn..

[40] Udani JP, Arrieta AF (2018). Efficient potential well escape for bi-stable Duffing oscillators. Nonlinear Dyn..

[41] Maree GJM (1997). Slow periodic crossing of a pitchfork bifurcation in an oscillating system. Nonlinear Dyn..

[42] Huang T, Dai L, Zhang H (2016). An approach combining periodicity ratio and secondary Poincaré map for characteristics diagnosis of nonlinear oscillatory systems. Nonlinear Dyn..

[43] Wawrzyński W (2018). Area of the unstable solution of rolling equation—jumps of the oscillations amplitude. J. KONES.

[44] Kawahara Y, Maekawa K, Ikeda Y (2012). A simple prediction formula of roll damping of conventional cargo ships on the basis of Ikeda’s method and its limitations. J. Shipping Ocean Eng..

[45] Wawrzyński W (2017). Predykcja składowej tłumienia kołysań bocznych statku dla stępek przechyłowych, porównanie skróconej i pełnej metody Ikedy. Zeszyty Naukowe Akademii Morskiej w Gdyni.

